# Comment on “The Effect of a Community-Based, Primary Health Care Exercise Program on Inflammatory Biomarkers and Hormone Levels”

**DOI:** 10.1155/2015/434218

**Published:** 2015-03-19

**Authors:** Bunyamin Koc, Hakan Sarman, Ismail Boyraz

**Affiliations:** ^1^Abant Izzet Baysal University, Physical Therapy and Rehabilitation Training and Research Hospital, Karacasu, 14020 Bolu, Turkey; ^2^Department of Orthopaedics and Traumatology, Abant Izzet Baysal University Faculty of Medicine, 14020 Bolu, Turkey

We read with great interest the recent paper by C. B. Papini et al. in which the authors examined “impact of a community-based exercise program in primary care on inflammatory biomarkers and hormone levels” in the 1-year quasiexperimental study [1]. The authors very clearly discussed the relation between exercise and inflammation. They concluded that community-based exercise program can result in a decrease or maintenance of inflammatory biomarkers after 1 year, and it has the potential to be a viable public health approach for chronic disease prevention. This study displayed that public health exercise intervention delivered in low-income communities has the potential to use a beneficial effect and improve or maintain inflammatory biomarkers profiles, supporting the prevention of chronic diseases.

The authors did not discuss exclusion criteria in this paper. However it is well established that any type of systemic inflammation, autoimmune disorders, and malignant or chronic illnesses may affect inflammatory biomarkers and hormone levels [2]. Also obesity is related to elevated serum levels of some inflammatory markers, such as leptin, TNF-*α*, and CRP [3, 4]. Because of high prevalence of these conditions, we believe that these situations may have a role in the results of the paper by C. B. Papini et al. [1].

In our opinion, future clinical studies assessment of considering these conditions may be helpful for exact results. We hope that bearing in mind these conditions would add to the value of the well-written paper of C. B. Papini et al. [1].
